# Engineered yeast with a CO_2_-fixation pathway to improve the bio-ethanol production from xylose-mixed sugars

**DOI:** 10.1038/srep43875

**Published:** 2017-03-06

**Authors:** Yun-Jie Li, Miao-Miao Wang, Ya-Wei Chen, Meng Wang, Li-Hai Fan, Tian-Wei Tan

**Affiliations:** 1 College of Life Science and Technology, Beijing University of Chemical Technology, Beijing, People’s Republic of China; 2 National Energy R&D Center for Biorefinery, Beijing, People’s Republic of China; 3 Beijing Key Laboratory of Bioprocess, Beijing, People’s Republic of China

## Abstract

Bio-ethanol production from lignocellulosic raw materials could serve as a sustainable potential for improving the supply of liquid fuels in face of the food-to-fuel competition and the growing energy demand. Xylose is the second abundant sugar of lignocelluloses hydrolysates, but its commercial-scale conversion to ethanol by fermentation is challenged by incomplete and inefficient utilization of xylose. Here, we use a coupled strategy of simultaneous maltose utilization and *in-situ* carbon dioxide (CO_2_) fixation to achieve efficient xylose fermentation by the engineered *Saccharomyces cerevisiae*. Our results showed that the introduction of CO_2_ as electron acceptor for nicotinamide adenine dinucleotide (NADH) oxidation increased the total ethanol productivity and yield at the expense of simultaneous maltose and xylose utilization. Our achievements present an innovative strategy using CO_2_ to drive and redistribute the central pathways of xylose to desirable products and demonstrate a possible breakthrough in product yield of sugars.

Fossil fuel depletion and environmental concerns drive the worldwide development of renewable fuel sources, with high focus on biofuels and biochemicals produced from alternative and sustainable raw materials^1^,^2^. Bio-ethanol, as typical example, can be produced by first, second and third generation processes depending on the use of hexoses, biomass cellulose-released sugars, or algae starch and cellulose, respectively^3^,^4^,^5^. Whereas first generation bio-ethanol processes are widely applied, they currently suffer from high production costs, low fossil fuel prices, and food-to-fuel competition^6^. The development of lignocellulosic raw materials is hence recognized as essential for a sustainable potential of bio-ethanol production^7^. Third generation bio-ethanol processes are at early stages of investigation, and only the algae left-over cake (after lipids extraction) should be considered, hence reversing into a second generation raw material of starch and cellulose. Xylose is the second most abundant sugar and consists of up to 35% of cellulosic sugars present in lignocellulosic biomass^8^. However, incomplete and inefficient conversion of xylose into bio-ethanol has hindered its commercial-scale processes.

Although xylose-fermenting microbes exist in nature, their ethanol production rates and tolerances are inferior to *Saccharomyces cerevisiae*^9^. Its endogenous pathway can catalyze xylulose to ethanol, but it cannot convert xylose to xylulose^10^. Metabolic engineering has thus been used to develop xylose-utilizing *S. cerevisiae*^11^,^12^,^13^. Generally, the xylose catabolism in engineered yeasts is mediated by a heterologously expressed fungal pathway consisting of xylose reductase (XR) and xylitol dehydrogenase (XDH)^14^, or by the bacterial xylose isomerase (XI)^15^ to covert xylose into xylulose. Compared with the XI-carrying yeasts, the XR-XDH-carrying strains had greater strengths on xylose consumption rate and ethanol productivity^16^. Moreover, co-expression of a xylulokinase (XK)^17^,^18^ and the key enzymes involved in the pentose phosphate pathway (PPP)^19^,^20^ with XR and XDH was able to further improve the utilization of xylose.

However, XR prefers nicotinamide adenine dinucleotide phosphate (NADPH) to nicotinamide adenine dinucleotide (NADH), while XDH uses only nicotinamide adenine dinucleotide (NAD^+^) as a cofactor^14^. Compared with the redox-neutral glycolysis^21^ for ethanol production from glucose, the engineered XR-XDH pathway in *S. cerevisiae* may lead to cofactor imbalance^22^. On the one hand, NADPH for XR is supplied by glucose-6-phosphate (G6P) metabolism through oxidative branch of PPP, but gluconeogenesis pathway for conversion of xylose to G6P is limited when *S. cerevisiae* grows on xylose^23^,^24^. On the other hand, catalysis of NAD^+^ to NADH by XDH usually facilitates the formation of by-products such as glycerol and xylitol^14^,^22^. Many efforts have been devoted to relieve the cofactor imbalance. Use of the mutant XR (mXR) that exhibits higher preference for NADH^25^ or the mutant XDH (mXDH) using nicotinamide adenine dinucleotide phosphate (NADP^+^)^26^ showed positive effects on xylose utilization. Besides, by co-expressing a heterogeneous acetate-consumption pathway^27^ or a carbon dioxide (CO_2_)-fixation pathway^28^ in the yeasts with XR and XDH, a decrease of by-products and an increase of ethanol from xylose were also observed since acetate acid and CO_2_ could be used as electric acceptor for NADH oxidation.

Therefore, the present research constructed a PRK-Rubisco module together with a mXR-XDH module into *S. cerevisiae*. Then, the resulted yeast strains were applied for xylose fermentation with simultaneous utilization of maltose and CO_2_ (Fig. 1). This strategy may have three major benefits: (i) The mXR-XDH module containing a mutant XR (R276H) preferring NADH, a XR, a XDH and a XK could improve the ethanol productivity and decrease the by-product accumulation. (ii) Catabolism of maltose could simultaneously supply energy and cofactors to meet the requirement for xylose metabolism. (iii) The CO_2_ fixation pathway may bypass the glucose repression on xylose catabolism inside the cell. The CO_2_ produced during fermentation can also form an appropriate atmosphere for Rubisco activity and a redox sink for improving the cofactor balance.

## Results

### Construction of xylose metabolic pathway in *S. cerevisiae*

We obtained a xylose-fermenting yeast strain YSX4 (Table 1) with co-expression of wild-type XR, mutant XR (R276H), XDH and XK in *S. cerevisiae* YS58. In previous work, the same xylose metabolic pathway has been used in an engineered *S. cerevisiae* DA24^29^. XR (R276H) was found to exhibit much higher preference for NADH whereas XR showed two-fold higher preference for NADPH^25^. Theoretically, co-expression of wild-type XR and mutant XR (R276H) could enable YSX4 with comparable XR activities towards NADPH and NADH.

The fermentation performance of the resulted YSX4 was evaluated under an oxygen-limited condition with xylose and glucose, or xylose and maltose as carbon source respectively (Table 2). We found that the xylose consumption rate of YSX4 (0.57 g/L/h) was similar with that of DA24 (0.53 g/L/h) on a glucose-xylose mixture, although the key enzymes involved in the xylose metabolic pathway in YSX4 were expressed by using a multi-copy plasmid, while in DA24, they were integrated in genome. Interestingly, compared with the glucose-xylose mixture, YSX4 exhibited a higher efficiency of xylose utilization (0.70 g/L/h) when grown on xylose and maltose, which is in agreement with previous results using cellobiose and xylose as carbon source^29^,^30^. Evidences showed that intracellular hydrolysis of cellobiose minimized glucose repression on xylose uptake, and simultaneous fermentation of cellobiose and xylose was capable of improving ethanol productivity when compared to fermentation with xylose only^30^. For both of maltose and cellobiose, disaccharides are formed from two glucose molecules. The difference is that glucose units are joined with an α (1 → 4) bond in maltose and a β (1 → 4) bond in cellobiose respectively. Therefore, we suspected that YSX4 could also co-ferment maltose and xylose, thus consumption of xylose by YSX4 was enhanced when cells were grown on maltose-xylose mixture.

### Functional expression of CO_2_-fixation pathway in *S. cerevisiae*

As a heterotrophic microorganism, *S. cerevisiae* intracellularly converts ribulose-5-phosphate (Ru5P) to glycerate-3-phosphate (G3P) through the non-oxidative branch of PPP without consumption of CO_2_. In contrast, many autotrophic microbes achieve this conversion by a two-step reaction using ribulose bisphosphate carboxylase-oxygenase (Rubisco) and phosphoribulokinase (PRK)^31^,^32^. PRK catalyzes Ru5P to ribulose-1,5-bisphosphate (Ru1,5BP), while Rubisco converts Ru1,5BP to G3P with CO_2_ fixation. There are four known forms of Rubisco in nature^33^. Form-I Rubisco (L_8_S_8_) is composed of eight large subunits and eight small subunits, of which the small subunits are able to concentrate surrounding CO_2_ to improve the reactivity of the large subunits. Form-II (L_8_) only contains eight large subunits. In previous works, the gene of *cbbM* from *Rhodospirillum rubrum* or *Thiobacillus denitrificans* coding for Form-II Rubisco and the gene of *sPRK* from *Spinacia oleracea* coding for PRK have already been successfully expressed in *S. cerevisiae*^28^,^34^. Interestingly, it was found that functional expression of *cbbM* in yeast depended on co-expression of a chaperone (*GroEL-GroES*) from *Escherichia coli*. In this work, we engineered *S. cerevisiae* YS58 with a Form-II based CO_2_ fixation system by co-expression of *cbbM, sPRK,* and *GroEL-GroES*, resulting in a strain named YSC111. Similarly, in another yeast strain called YSC222, *cbbL1-cbbS1* (Form-I Rubisco gene) and *cfxP1* (PRK gene) from *Ralstonia eutropha* H16^35^ constructed a Form-I based CO_2_ fixation system. To ensure the functionality of Form-I Rubisco, an endogenous chaperone (*Hsp60-HSP10*) of *S. cerevisiae* YS58 was simultaneously over-expressed.

**Figure 2:**
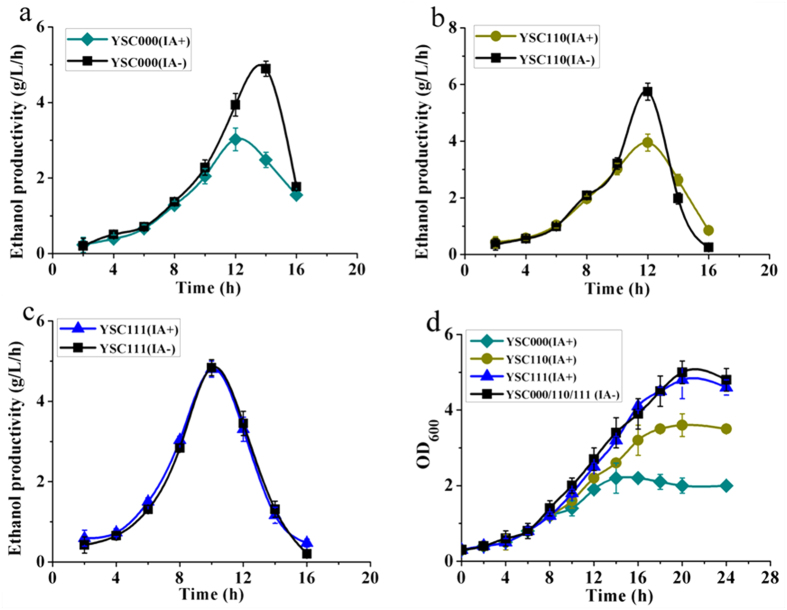
Fermentation profiles of YSC000, YSC110 and YSC111 in YP medium containing 70 g/L glucose with IA (IA+) or without IA (IA-) addition.

To test whether the CO_2_-fixation pathway was workable in the engineered *S. cerevisiae*, iodoacetate (IA) was used to inhibit the activity of glyceraldehyde-3-phosphate dehydrogenase (GAPDH), thus repressing the cellular glycolysis. After addition of IA to 0.19 g/L (~0.001 mol/L), the ethanol productivity of YSC000 or YSC110 decreased sharply (Fig. 2a,b), but no negative effects on YSC111were observed (Fig. 2c). YSC111 also showed similar growth profiles with or without IA addition (Fig. 2d). The ethanol yields of YSC222 and YSC111 were slightly affected by IA, but the ethanol yields of YSC220 and YSC110 lacking of chaperones decreased by 27% (Table S1), indicating that chaperones were essential for functional expression of both Form-I and Form-II Rubisco. We also found that the performance of the engineered CO_2_-fixation pathway would be enhanced with increase of glucose concentrations (Fig. S1), which was probably due to more surrounding CO_2_ present.

### Effects of CO_2_ fixation on xylose utilization

**Figure 3:**
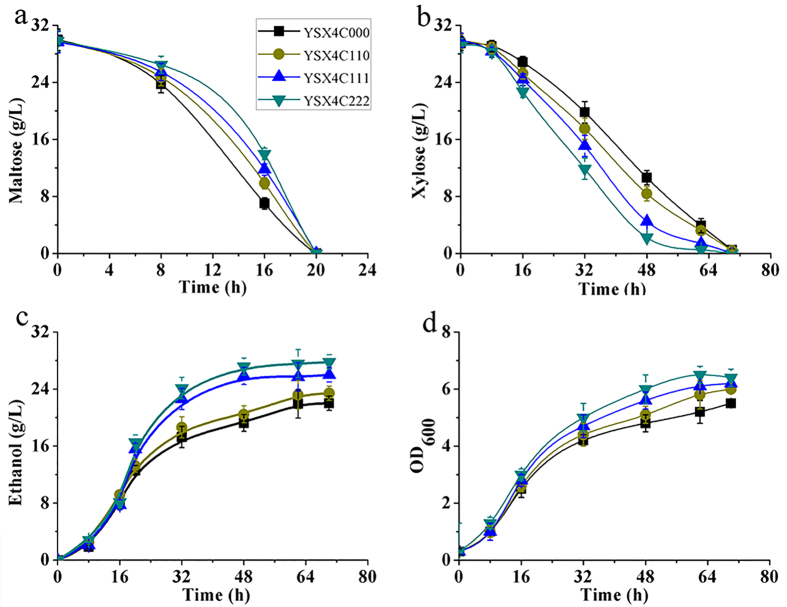
Fermentation profiles of YSX4C000, YSX4C110, YSX4C111 and YSX4C222 in YP medium containing 30 g/L maltose and 30 g/L xylose.

The functional CO_2_-fixation pathway was constructed in the engineered *S. cerevisiae* YSX4, resulting in YSX4C111 and YSX4C222. YSX4C222 exhibited the highest xylose consumption rate, ethanol productivity and strain growth rate (Fig. 3). Compared with YSX4C000, the rate of xylose consumption elevated from 0.54 g/L/h to 0.70 g/L/h by using YSX4C222 (Table 3), while both of the final ethanol level and cell density got a over 25% increase (Fig. 3c,d).

Then, YSX4C111 and YSX4C222 were applied in the medium containing 70 g/L maltose and 40 g/L xylose. As shown in Table 2, the xylose consumption rates of YSX4C111 and YSX4C222 arrived to 0.97 and 1.1 g/L/h respectively. The total sugar consumption rate and the ethanol yield of YSX4C222 reached 3.1 g/L/h and 0.47 g/g sugars, which was 63% and 15% higher than YSX4C000. YSX4C222 cell extract also showed the highest carboxylation activity (Fig. S2). These findings suggested that the Form-I based CO_2_ fixation system was preferred in facilitation of the xylose metabolism.

### Relative quantification of heterotrophic CO_2_ fixation

To evaluate the strength of the CO_2_ flux in YSX4C111 and YSX4C222, a reported metabolic flux index, MFI_h-CO2_^36^, was employed to indicate the metabolic flux ratio between the CO_2_-fixation and the PPP-based pathways. The G3P generated by these two pathways was differentiated by using ^13^C-labeled CO_2_ and unlabeled sugars to obtain the MFI_h-CO2_ value.

As illustrated in Fig. S3, we assumed that *a* mole of glyceraldehyde-3-phosphate (GADP) was metabolized to G3P, while *b* mole of ^13^CO_2_ from NaH^13^CO_3_ and ^12^CO_2_ from sugars was fixed to G3P within a given time period. Moreover, we assumed that *c* mole of unlabeled G3P and *d* mole of ^13^C-G3P were channeled into the downstream metabolism. Fermentation experiments were performed using 10 g/L maltose and 20 g/L xylose with 100 mM NaH^13^CO_3_ and cell density of OD_600_ = ~0.1. The amounts of 13C-labeled and unlabeled G3P were measured by LC-MS/MS. *S. cerevisiae* YS58 was cultivated in a medium free of any carbon isotope to determine the ratio of ^13^C-G3P to the unlabeled G3P as the basal isotopic level, which was about 3.1%. The actually detected molar amount of ^13^C-G3P (y) can be calculated by Eq. (1), while the actually detected unlabeled G3P (x) can be calculated by Eq. (2).


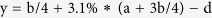



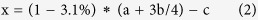


Under a metabolic steady-state, the relationship of d, c, x and y is shown in Eq. (3).


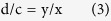


Therefore, the value of MFI_h−CO2_ can be calculated by Eq. (4).


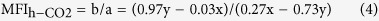


**Figure 4:**
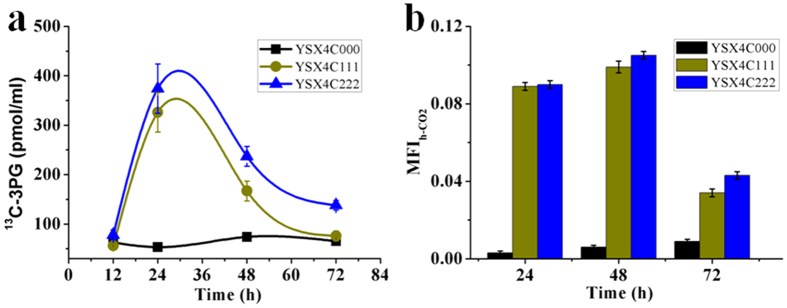
Intracellular ^13^C-G3P contents (**a**) and MFI_h-CO2_ values (**b**) of YSX4C000, YSX4C111 and YSX4C222.

As shown in Fig. 4a, ^13^C-G3P amount in YSX4C000 did not change during fermentation. In contrast, a significant increase of ^13^C-G3P was observed in YSX4C111 and YSX4C222. These results demonstrated that CO_2_ was able to be incorporated into G3P through the engineered CO_2_-fixation pathways. The MFI_h-CO2_ values of these strains at different times were calculated to evaluate the relative CO_2_ flux (Fig. 4b), and the results indicated that over 8% of Ru5P from xylose was converted to G3P through the CO_2_-fixation pathway in YSX4C111 or YSX4C222. More interestingly, the higher levels of ^13^C-G3P and MFI_h-CO2_ in YSX4C222 than in YSXC111 suggested that the Form-I based system was more efficient than Form-II in *S. cerevisiae*.

### Determination of the CO_2_-fixation rate in the engineered yeasts

The carbon balance of wild-type *S. cerevisiae*^37^ indicated that 96% carbon of the consumed sugars (C_consumed sugars_) was incorporated into biomass (C_biomass_), CO_2_, ethanol (C_ethanol_), xylitol (C_xylitol_), glycerol (C_glycerol_) and Acetate (C_acetate_). Therefore, the carbon of CO_2_ fixed by YSX4C111 or YSX4C222 (C _fixed CO2_) can be calculated using Eq. (5). C_CO2 with biomass_ and C_CO2 with ethanol_ represent the CO_2_ from cell respiration and from ethanol biosynthesis respectively.


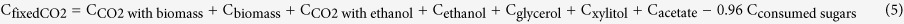


We assume that C_CO2 with biomass_ is in direct proportion to C_biomass_. Therefore, Eq. (5) can be transformed into Eq. (6).


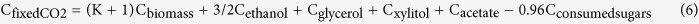


The carbon balance of YSX4C000 is shown as Eq. (7).


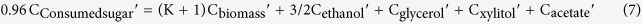


Substitution of K in Eq. (6) into Eq. (7), thus


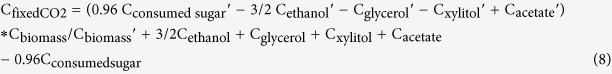


Fermentation data of YSX4C000, YSX4C111 or YSX4C222 on 70 g/L maltose and 40 g/L xylose are summarized in Table 3. The results showed that our yeasts were able to fix CO_2_ at a rate of 336.6–436.3 mg CO_2_/L/h, significantly exceeding the natural or the engineered microbes (5.8 to 147.0 mg CO_2_/L/h) in previous reports^36^ (Table S2).

## Discussion

Currently, xylose fermentation with the engineered *S. cerevisiae* is inferior to glucose fermentation by the wild-type *S. cerevisiae*. Besides cofactor imbalance of the engineered XR-XDH pathway, supply of NADPH was limited because expression of the enzymes involved in gluconeogenesis in *S. cerevisiae* cannot be upregulated on xylose. Some rate-limiting enzymes in the non-oxidative branch of PPP need to be over-expressed to enhance the xylose downstream metabolic flux. Here, we engineered *S. cerevisiae* to achieve efficient bio-ethanol production from maltose and xylose with CO_2_ fixation. NADH could be reduced through the mXR and CO_2_-fixation pathway, while the NADPH for the wild-type XR and the adenosine triphosphate (ATP) for activating Ru5P to Ru1,5BP used in Rubisco reaction were provided by co-fermentation of maltose and xylose.

Xylose can enter the cell by facilitated diffusion through some yeast hexose transporters (HXTs)^38^. For engineered XR-XDH-carrying yeasts, intracellular xylose conversion is only slightly affected by catabolism of intracellular glucose, but xylose transport is strongly inhibited by extracellular glucose^39^. The engineered xylose transporters by non-rational mutagenesis or rational design have been used to improve simultaneous utilization of xylose and glucose^40^,^41^. However, the specific xylose transporters without glucose inhibition have still not been developed^42^. On the other hand, introduction of a cellodextrin transport (*CDT-1*) from *Neurospora crassa* into the yeast with intracellular β-glucosidase (*GH1-1*) achieved simultaneous utilization of cellobiose and xylose and showed synergistic effects due to sufficient NADPH supply^29^,^30^. But in yeast, β-glucosidase (*GH1-1*) can convert cellobiose to cellodextrins (cellotriose, cellotetraose) by its transglycosylation activity, while cellodextrin transport (*CDT-1*) can transport cellodextrins and glucose into the medium. This problem resulted in low ethanol yield and productivity from cellobiose and xylose. In this study, we employed another disaccharide, maltose, which can be utilized as efficiently as glucose by the wild-type yeast, to facilitate xylose metabolism and achieved the synergistic effects.

Heterotrophic Form-I and Form-II based CO_2_ fixation systems were successfully expressed in *S. cerevisiae*. We observed that the engineered yeast YSX4C111 and YSX4C222 grew better and exhibited higher ethanol productivity than YSX4C000. The results suggest that additional ATP consumption by implementing the CO_2_-fixation pathway had a less negative effect on cell growth than the beneficial effect that the pathway might bring during co-fermentation of xylose and maltose. With increase of sugar concentrations in fermentation, the engineered yeasts expressing CO_2_ fixation pathway showed better ethanol productivity. The possible reason was more CO_2_ production from sugars increased environmental CO_2_ and HCO_3_^−^ concentrations so that improved the carboxylation activity of Rubisco under oxygen-limited condition. The same effects were also observed by supplementation of external CO_2_^34^. The activity of Rubisco could further be optimized through expressing heterogeneous carbonic anhydrase (CA)^36^, which catalyzes the reversible conversion of CO_2_ and HCO_3_^−^, or constructing a microcompartment for Rubisco^43^, similar to the semi-permeable carboxysome in *cyanobacteria*.

At present, low efficiency of photosynthetic processes and electro-synthetic processes using photoautotrophic and chemoautotrophic microbes are limited in their growths and productivities^32^,^44^. Differing from autotrophic microbes, heterotrophic microbes such as *S. cerevisiae*, which has very short doubling time, have been widely applied in industrial production of bio-chemicals. The CO_2_ fixation at the expense of sugars in heterotrophic *S. cerevisiae* presented high CO_2_-fixation rates and product yields. The construction of a functional CO_2_-fixation pathway for efficient ethanol production from xylose and maltose in *S. cerevisiae* would create a foundation for other biofuels and chemicals production from lignocellulosic biomass.

## Methods

### Strains, plasmids and media

*Escherichia coli* Trans10 (TransGen Biotech) was used for genetic manipulation. The plasmids and yeasts used in this work were summarized in Table 1. Synthetic complete (SC)-Ura-Leu minimal medium contains 0.67% yeast nitrogen base with ammonium sulfate and without amino acids (YNB), 2% glucose, 0.005% histidine and 0.01% tryptophan. SC-Leu or SC-Ura minimal medium contains 0.67% YNB, 2% glucose, 0.005% histidine, 0.01% tryptophan, 0.005% leucine or 0.01% Uracil. SC-Ura-Leu, SC-Ura and SC-Leu medium contains 0.67% YNB, 2% glucose, 0.01% (adenine, arginine, cysteine, lysine, threonine, tryptophan) and 0.005% (aspartic acid, histidine, isoleucine, methionine, phenylalanine, proline, serine, tyrosine, valine), while appropriate leucine and uracil addition when required. YP medium contains 1% yeast extract and 2% peptone.

### Construction of xylose and CO_2_-fixaiton pathway

The gene expression cassettes of xylose pathway were constructed. The genes of *XYL1* (Gene ID: 4839234, coding for XR) and *XYL2* (Gene ID: 4852013, coding for XDH) from *S. stipitis* CBS6054 (Laboratory-stored), and *XKS1* (Gene ID: 853108, coding for XK) from *S. cerevisiae* YS58 (laboratory-stored) were amplified by using the primers in Table S3. Obtained gene fragments were inserted between promoters and terminators individually in the recombinant pUC19 as shown in Table S4. The mutant *XYL1* (A826C, G827A, A828C) coding for XR (R276H) was amplified from pUC19-*TEF2p-XYL1-TPI1t* using point mutant primers (R276H-F and R276H-R) (Table S3), and then mutant XYL1 fragment was cloned and inserted into pUC19*-TEF1p-mXYL1-PGIt* (Table S4).

The genes of *sPRK* (GenBank: X07654.1) and *cfxP1* (Gene ID: 4456348), *cbbM* (GenBank: L37437.2) and *cbbL1-cbbS1* (GenBank: U20584.1), and *GroEL-GroES* (Gene ID: 948665) and *HSP60-HSP10* (Gene ID: 850963, no mitochondrion signal peptide) were chosen for construction of the CO_2_-fixation pathway. Except *HSP60-HSP10,* other genes were codon-optimized by Jcat^45^ (http://www.jcat.de/) and synthesized in KLSBE (Key Laboratory of Systems Bioengineering, Ministry of Education, China). The synthetic gene fragments were linked into pEASY-Blunt with the promoters and terminators (Table S4). The gene expression cassettes were linked into pRS425 or YCplac33 vectors to generate yeast expression plasmids (Table 1). *S. cerevisiae* transformants were selected on SC-Ura-Leu, SC-Leu or SC-Ura minimal medium.

### Fermentation experiments

The engineered yeast YSX4 was pre-cultured in SC-Leu medium. YSC000, YSC110, YSC111 and YSC222 were pre-cultured in SC-Ura medium. YSX4C000, YSX4C110, YSX4C111 and YSX4C222 were pre-cultured in SC-Ura-Leu medium. After washing with distilled water, the cells were inoculated into 200 mL YP medium with appropriate sugars (maltose, glucose or xylose) in 500 mL flasks. All fermentation experiments were carried out at 30 °C and 200 rpm under oxygen-limited conditions. The initial cell density was adjusted to an OD_600_ (optical density at 600 nm) of ~0.5. Rubisco activity was determined through Rubisco Carboxylation Activity Assay Kit (GENMED, Shanghai) by UV-visible Spectrophotometer SU-2000.

### IA inhibition assay

The cells of YSC000, YSC110, YSC111 and YSC222 were inoculated into 200 mL YP medium with 70 g/L glucose in 500 mL flasks. The initial cell density was adjusted to an OD_600_ of ~0.1. IA was added to the media at 8 h. Ethanol concentration and OD_600_ were detected in whole fermentation process.

### Analytical methods

Cell growth was monitored using UV-visible Spectrophotometer SU-2000 (OnLab Instruments). Maltose, glucose, xylose, xylitol, glycerol, acetate and ethanol were quantified by high performance liquid chromatography (Agilent 1200 Series HPLC system) equipped with a refractive index detector (Shimadzu, Japan) and an Bio-Rad Aminex HPX-87H organic acid analysis column (7.8 × 300 mm) which was maintained at 50 °C and used 0.05 mM sulfuric acids as mobile phase. The sample injection volume was 10 μL and the flow rate was 0.6 mL/min. Metabolites was detected by liquid chromatography-mass spectrometry/mass spectrometry system (Agilent 6460 series LC-MS/MS system) with Agilent XDC18 column (5 uM, 150 mm × 4.6 mm)^36^. Di-*n*-butylammonium acetate (DBAA) and NaH^13^CO_3_ were purchased from Sigma-Aldrich. Methanol was purchased from Fisher Scientific. The mobile phase was the mixture of solution A (water with 5 mM DBAA) and solution B (methanol with 5 mM DBAA) at the gradient shown in Table S6. The flow rate was 0.6 mL/min. The injection volume was 50 μL and the column temperature was 40 °C. The negative ion and selected multiple reactions monitoring (MRM) mode were used for MS detection. All experiments were conducted at least in triplicate, and the error bars in the figures denote the standard deviation from the means of independent experiments.

## Additional Information

**How to cite this article:** Li, Y.-J. *et al*. Engineered yeast with a CO_2_-fixation pathway to improve the bio-ethanol production from xylose-mixed sugars. *Sci. Rep.* **7**, 43875; doi: 10.1038/srep43875 (2017).

**Publisher's note:** Springer Nature remains neutral with regard to jurisdictional claims in published maps and institutional affiliations.

## Supplementary Material

Supplementary Information

## Figures and Tables

**Figure 1:**
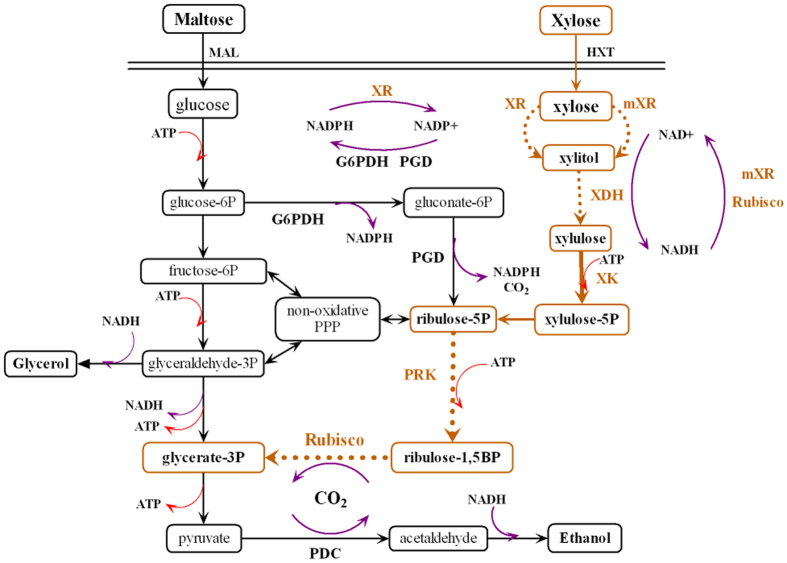
Engineered *S. cerevisiae* for co-utilization of xylose, maltose and CO_2_. MAL: maltose transporter; HXT: hexose transporter; XR: xylose reductase; mXR: xylose reductase with the mutant of R276H; XDH: xylitol dehydrogenase; XK: xylulokinase; PRK: phosphoribulokinase; Rubisco: ribulose bisphosphate carboxylase-oxygenase; PPP: pentose phosphate pathway; G6PDH: glucose 6-phosphate dehydrogenase; PGD: 6-phosphogluconate dehydrogenase; PDC: pyruvate decarboxylase; P: phosphate; BP-bisphosphate; ATP: adenosine triphosphate.

**Table 1:** Plasmids and strains used in this work.

Plasmids and strains	Description
Plasmids	
pRS425	*LEU2*, a multi-copy plasmid
pPS425*-mXYL1-XYL2-XKS1-XYL1*	Expression of XR (R276H), XDH, XK and XR through pPS425
YCplac33	*URA3*, a single-copy plasmid
YCplac33*-cbbM-sPRK*	Expression of cbbM and sPRK through YCplac33
YCplac33*-cbbM-cfxP1*	Expression of cbbL1-cbbS1 and PRK through YCplac33
YCplac33*-cbbM-sPRK-GroEL-GroES*	Expression of cbbM, sPRK and GroEL-GroES through YCplac33
YCplac33-*cbbL1-cbbS1-cfxP1-HSP60-HSP10*	Expression of cbbL1-cbbS1, PRK and HSP60-HSP10 through YCplac33
Strains	
YS58	*MATa, leu2, his3,ura3, trp1*
YSX4	YS58 harboring pPS425*-mXYL1-XYL2-XKS1-XYL1*
YSC000	YS58 harboring YCplac33
YSC110	YS58 harboring YCplac33*-cbbM-sPRK*
YSC111	YS58 harboring YCplac33*-cbbM-sPRK-GroEL-GroES*
YSC220	YS58 harboring YCplac33*-cbbM-cfxP1*
YSC222	YS58 harboring YCplac33*-cbbM-cfxP1- HSP60-HSP10*
YSX4C000	YS58 harboring pPS425*-mXYL1-XYL2-XKS1-XYL1* and YCplac33
YSX4C110	YS58 harboring pPS425*-mXYL1-XYL2-XKS1-XYL1* and YCplac33*-cbbM-sPRK*
YSX4C111	YS58 harboring pPS425*-mXYL1-XYL2-XKS1-XYL1* and YCplac33*-cbbM-sPRK-GroEL-GroES*
YSX4C220	YS58 harboring pPS425*-mXYL1-XYL2-XKS1-XYL1* and YCplac33-*cbbL1-cbbS1-cfxP1*
YSX4C222	YS58 harboring pPS425*-mXYL1-XYL2-XKS1-XYL1* and YCplac33-*cbbL1-cbbS1-cfxP1-HSP60-HSP10*

**Table 2:** Fermentation performances of the engineered yeasts in this work.

Sugars (g/L)	Strains	Y_Eth_	V_Eth_	R_Xyl_	R_Glc/Mal_	R_Total_	Reference
**Xylose (40)**	YSX4	0.35	0.12	0.33	—	—	This study
**Glucose/Xylose**	DA24	0.39	0.74	0.53	—	1.5	33
**(70/40)**	YSX4	0.38	0.60	0.57	3.8	1.6	This study
**Maltose/Xylose (70/40)**	YSX4	0.42	0.88	0.76	3.2	2.1	This study
**Glucose (70)**	YSX4	0.45	1.8	—	3.9	—	This study
**Maltose (70)**	YSX4	0.44	1.7	—	3.5	—	This study
**Maltose/Xylose (70/40)**	YSX4C000	0.41	0.9	0.69	2.9	1.9	This study
	YSX4C110	0.41	1.0	0.73	2.8	2.0	This study
	YSX4C111	0.46	1.3	0.97	2.4	2.8	This study
	YSX4C222	0.47	1.5	1.1	2.2	3.1	This study
**Maltose/Xylose (30/30)**	YSX4C000	0.37	0.32	0.54	2.1	0.86	This study
	YSX4C110	0.39	0.38	0.58	2.0	0.93	This study
	YSX4C111	0.43	0.50	0.68	1.8	1.2	This study
	YSX4C222	0.46	0.57	0.70	1.6	1.3	This study

Y_Eth_: ethanol yield (g ethanol/g sugar): V_Eth_: ethanol productivity (g/L/h); R_Xyl_: xylose consumption rate (g/L/h); R_Glc/Mal_: glucose or maltose consumption rate (g/L/h); R_Total_: total sugar consumption rate (g/L/h).

**Table 3:** The CO_2_-fixation rates of engineered YSX4C000, YSX4C111 and YSX4C222.

Sugars (g/L)	Strains	C_Eth_	C_Xyl_	C_Gly_	C_Xylit_	C_Acet_	DCW	R_CO2_
Maltose/Xylose (70/40)	YSX4C000	38.9 ± 0.5	15.2 ± 0.5	3.2 ± 0.3	2.8 ± 0.1	0.2 ± 0.1	5.2 ± 0.2	—
	YSX4C111	48.2 ± 0.5	5.2 ± 0.5	2.5 ± 0.2	2.4 ± 0.2	0.1 ± 0.1	5.7 ± 0.3	336.6 ± 1.5
	YSX4C222	51.9 ± 0.5	0.3 ± 0.3	1.6 ± 0.2	2.0 ± 0.1	0.1 ± 0.1	6.4 ± 0.3	436.3 ± 3.0

C_Eth_: ethanol concentration (g/L); C_Xyl_: Residual xylose concentration (g/L); C_Gly_: glycerol concentration (g/L); C_Xylit_: xylitol ethanol concentration (g/L); C_Acet_: acetate concentration (g/L); DCW: Dry Cell Weight (g/L); R_CO2_: CO_2_-fixation rate (mg CO_2_/L/h).
