# Supplementary material for: Engineered yeast with a CO2-fixation pathway to improve the bio-ethanol production from xylose-mixed sugars
Source: Sci Rep. 2017 Mar 6;7:43875. doi: 10.1038/srep43875 (PMC5338314; doi:10.1038/srep43875)
Supplement: Supplementary Information [file srep43875-s1.doc]

# Supplementary Information

**Engineered yeast with a CO_2_-fixation pathway to improve the bio-ethanol production from xylose-mixed sugars**

Yun-Jie Li^1,2,3^, Miao-Miao Wang^1,2,3^, Ya-Wei Chen^1,2,3^, Meng Wang^1,2,3^, Li-Hai Fan^1,2,3^*, Tian-Wei Tan^1,2,3^,*

^1^College of Life Science and Technology, Beijing University of Chemical Technology, Beijing, People’s Republic of China.

^2^National Energy R&D Center for Biorefinery, Beijing, People’s Republic of China.

^3^Beijing Key Laboratory of Bioprocess, Beijing, People’s Republic of China.

*Corresponding authors: Li-Hai Fan, Tian-Wei Tan

**
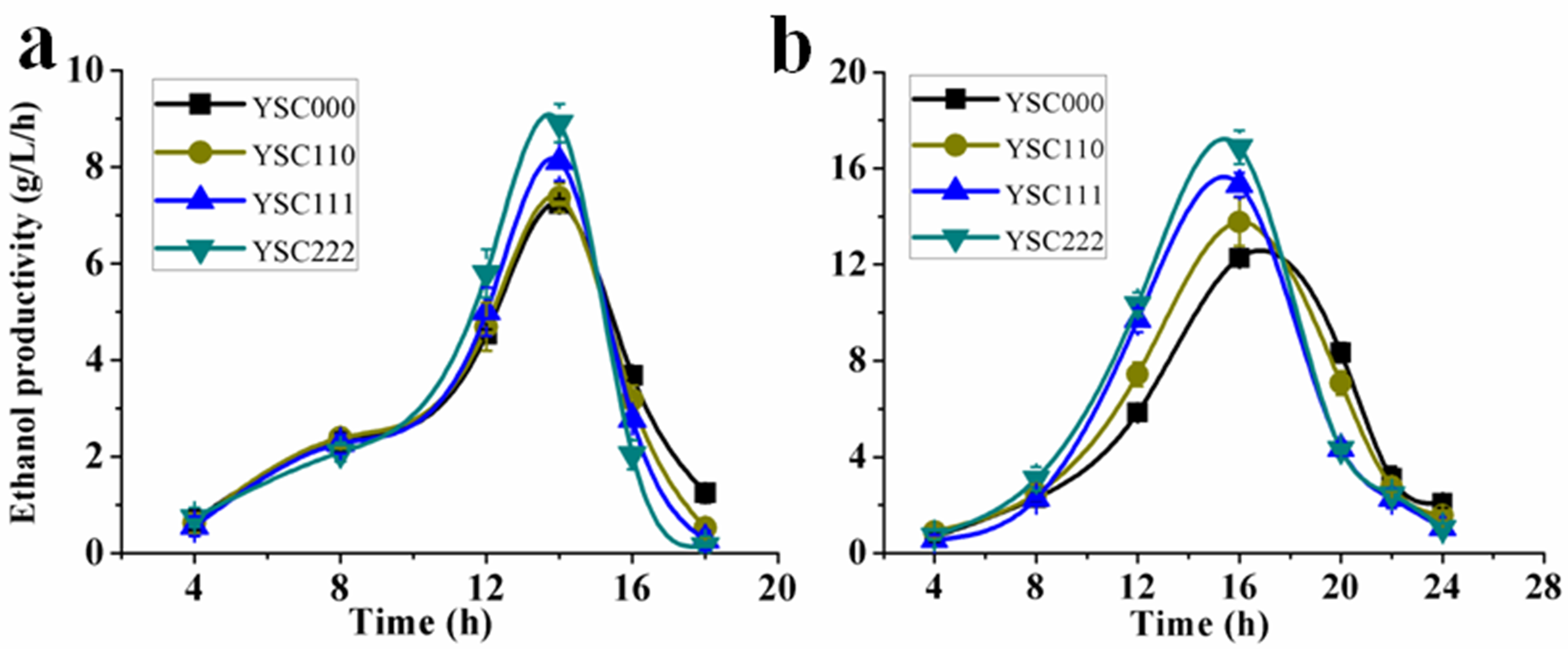
**

**Fig. S1** Ethanol productivities of YSC000, YSC110, YSC111 and YSC222 in YP medium containing100 (**a**) or 160 (**b**) g/L glucose.


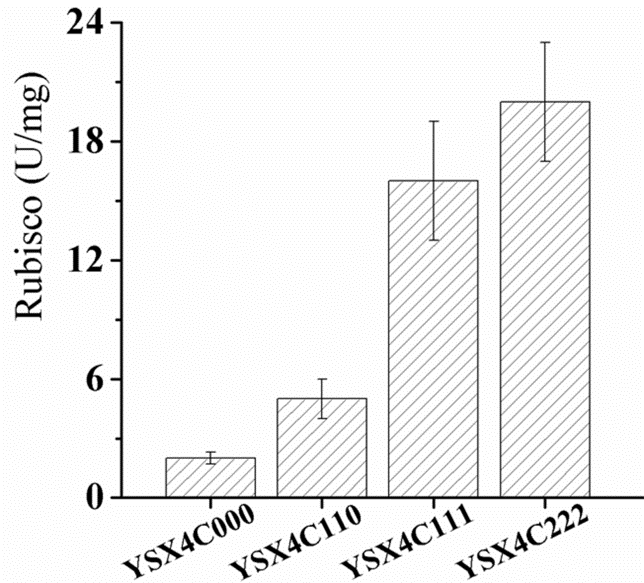


**Fig. S2** Rubisco carboxylation activity in *S. cerevisiae* strains expressing different synthetic constructs.


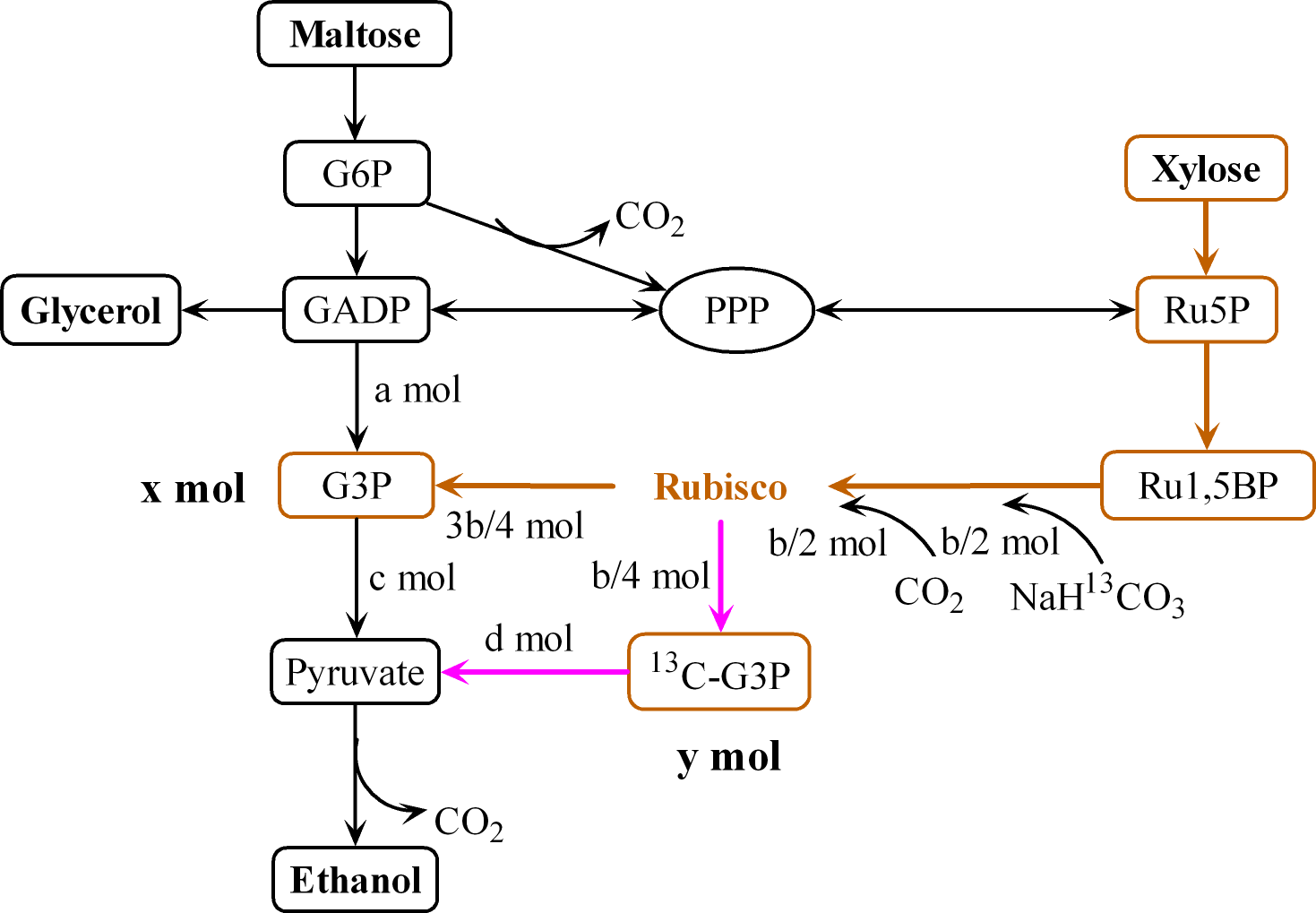


**Fig. S3** Relative quantitative analysis of CO_2_ fixation. G6P: glucose-6-phosphate; GADP: glyceraldehyde-3-phosphate; PPP: pentose phosphate pathway; G3P: glycerate-3-phosphate; Ru5P: ribulose-5-phosphate; Ru1,5BP: ribulose-1,5-bisphosphate.

**Table S1** Ethanol yield of YSC000, YSC110, YSC111 and YSC222 in YP medium containing 70 g/L glucose with or without IA addition

| **Strains** | **Ethanol yield (g/g) with IA** | **Ethanol yield (g/g) without IA** |
| --- | --- | --- |
| **YSC000** | 0.31 | 0.44 |
| **YSC110** | 0.32 | 0.43 |
| **YSC111** | 0.42 | 0.45 |
| **YSC220** | 0.31 | 0.44 |
| **YSC222** | 0.45 | 0.47 |

**Table S2** A summary of the CO_2_-fixation rates of autotrophic and heterotrophic CO_2_-fixing microbes

| **Strians** | **Species** | **CO_2_-fixation Rate**  **(mg/L/h)** | **Biomass**  **(g DCW/L)** | **Specific CO_2_-fixation rate**  **(mg/g DCW/L)** | **CO_2_ concentration (%)** |
| --- | --- | --- | --- | --- | --- |
| **Autotrophic microbes** | |  |  |  |  |
| ***Microalgae*** | *Chlorella vulgaris* | 53.0 | 5.7 | 9.3 | 5 |
|  | *Scenedesmus sp. NIER-10060* | 25.5 | 2.7 | 9.4 | 15 |
|  | *Botryococcus braunii SAG-30.81* | 21.0 | 3.1 | 6.8 | 10 |
| ***Cyanobacteria*** | *Anabaena sp. ATCC 33047* | 60.4 | 2.7 | 22.4 | 0.03 |
|  | *Aphanothece microscopica* | 109.0 | 5.1 | 21.4 | 15 |
| ***Non-green algae*** | *Phaeodactylum tricornutum* | 147.0 | 6.2 | 23.7 | 40 |
| **Heterotrophic microbes** | | | | | |
| ***E. coli*** | *E. coli* JB | 5.8 | 6.1 | 0.95 | 0.03 |
|  | *BL21(DE3)*/PET-RBC-PRK | 13.3 | 0.82 | 16.2 | 5 |
|  | *BL21(DE3)*/PET-RBC-PRK-CA | 19.6 | 0.87 | 22.5 | 5 |
| ***S.cerevisiae*** | YSX4C111 | 336.6 | 5.7 | 59.1 | 0 |
|  | YSX4C222 | 436.3 | 6.4 | 68.2 | 0 |

**Table S3 Primers used for construction of xylose gene cassettes**

| **Primers** | **Forward** | **Reverse** |
| --- | --- | --- |
| ***XYL1*** | CGAGCTCATGCCTTCTATTAAGTTGAACTCTGG | GCTCTAGATTAGACGAAGATAGGAATCTTGTCCC |
| ***XYL2*** | CGAGCTCATGACTGCTAACCCTTCCTTGGT | ACGCGTCGACTTACTCAGGGCCGTCAATGA |
| ***XKS1*** | GGAATTCATGTTGTGTTCAGTAATTCAGAGACAG | GGAATTCTTAGATGAGAGTCTTTTCCAGTTCGC |
| **R276H** | ACTGTCCCA**CAC**TTGTTGGAAAACA | TCCAACAA**GTG**TGGGACAGTGTTG |

**Table S4** The gene cassettes of xylose and CO_2_-fixation pathway

| **Plasmids** | **Description** | **Reference** |
| --- | --- | --- |
| pUC19*-PGK1p-αFactor-CYC1t* | Insert of *PGK1* promoter, α-factor, and *CYC1* terminator in pUC19. | [1] |
| pUC19*-TEF1p-αFactor-PGIt* | Insert of *TEF1* promoter, α-factor, and *PGI* terminator in pUC19. | [1] |
| pUC19*-TEF2p-αFactor-TPI1t* | Insert of *TEF2* promoter, α-factor, and *TPI*1 terminator in pUC19. | [1] |
| pRS424*-HXT7p-CDT-HXT7t* | Insert of *HXT7* promoter, *CDT*, and *HXT7* terminator in pRS424. | [2] |
| pUC19*-TEF2p-XYL1- TPI1t* | *XYL1* expression cassette. | This work |
| pUC19*-TEF1p-mXYL1-PGIt* | m*XYL1* expression cassette. | This work |
| pUC19*-PGK1p-XYL2-CYC1t* | *XYL2* expression cassette. | This work |
| pRS424*-HXT7p-XKS1-HXT7t* | *XKS1* expression cassette. | This work |
| pEASY-Blunt*-PGI1p-sPRK-CYC1t* | Insert of *PGI1* promoter, *sPRK*, and *CYC1* terminator in pEASY-Blunt. *sPRK* expression cassette. | This work |
| pEASY-Blunt*-PGI1p-cfxP1-CYC1t* | Insert of *PGI1* promoter, *cfxP1*, and *CYC1* terminator in pEASY-Blunt. | This work |
| pEASY-Blunt*-TDH3p-cbbM-TEF2t* | Insert of *TDH3* promoter, *cbbM*, and *TEF2* terminator in pEASY-Blunt. | This work |
| pEASY-Blunt*-TDH3p-cbbL1-TEF2t* | Insert of *TDH3* promoter, *cbbL1*, and *TEF2* terminator in pEASY-Blunt. | This work |
| pEASY-Blunt*-CCW12p-cbbS1-ADH1t* | Insert of *CCW12* promoter, *cbbS1*, and *ADH1* terminator in pEASY-Blunt. | This work |
| pEASY-Blunt*-FBA1p-GroEL-TDH2t* | Insert of *FBA1* promoter, *GroEL*, and *TDH2* terminator in pEASY-Blunt. | This work |
| pEASY-Blunt*-GPM1p-GroES-GPDt* | Insert of *GPM1* promoter, *GroES*, and *GPD* terminator in pEASY-Blunt. | This work |
| pEASY-Blunt*-FBA1p-HSP60-TDH2t* | Insert of *FBA1* promoter, *HSP60* and *TDH2* terminator in pEASY-Blunt. | This work |
| pEASY-Blunt*-GPM1p-HSP10-GPDt* | Insert of *GPM1* promoter, *HSP10*, and *GPD* terminator in pEASY-Blunt. | This work |

**Table S5** Primers used for shifting gene cassettes into pRS425 orYCplac33

| **Name** | **Sequence** |
| --- | --- |
| **XYL1(R276H)-F (SmaI)** | TCCCCCGGGATCGATATAGCTTCAAAATGTTTCTACT |
| **XYL1(R276H)-R(SmaI/NdeI)** | TCCCCCGGGCATATGGGTATACTGGAGGCTTCAT |
| **XYL2-F(SacII)** | TCCCCGCGGACGCACAGATATATAACATCTGCAT |
| **XYL2-R(SacII)** | TCCCCGCGGGCAATTAAAGCCTTCGAGCG |
| **XYL1-F(NdeI)** | GGAATTCCATATGGGGCGCCATAACCAAGGTATCTATA |
| **XYL1-R(NdeI)** | GGAATTCCATATGCTATATAACAGTTGAAATTTGGATA |
| **XKS1-F (NotI)** | ATAAGAATGCGGCCGCACTTCTCGTAGGACAATTTCG |
| **XKS1-R (NotI)** | ATAAGAATGCGGCCGCATAACTGACTCATTAGACACT |
| **cbbM-F (SmaI)** | TCCCCCGGGTCAGTTCGAGTTTATCATTATCAATA |
| **cbbM-R (SmaI)** | TCCCCCGGGGATGAGGCCGTCTTTTGTTGAT |
| **cbbL1-cbbS1-F (SmaI)** | TCCCCCGGGTCAGTTCGAGTTTATCATTATCAATA |
| **cbbL1-cbbS1-R (SmaI)** | TCCCCCGGGGATGAGGCCGTCTTTTGTTGAT |
| **sPRK-F (SalI)** | ACGCGTCGACGGTGGGTGTGGGTGTATTGGAT |
| **sPRK-R (SalI)** | ACGCGTCGACGCAAATTAAAGCCTTCGAGCGTCCCAA |
| **cfxP1-F (SalI)** | ACGCGTCGACGGTGGGTGTGGGTGTATTGGAT |
| **cfxP1-F (SalI)** | ACGCGTCGACGCAAATTAAAGCCTTCGAGCGTCCCAA |
| **GroEL-F(KpnI)** | GGGGTACCCTTCATGCCTCCAACGGCTACTAT |
| **GroEL-R(KpnI)** | GGGGTACCGCGAAAAGCCAATTAGTGTG |
| **GroES-F(SphI)** | ACATGCATGCGCTACGCAGGCTGCACAATTA |
| **GroES-R(SphI)** | ACATGCATGCGGAATCTGTGTATATTACTGCATCTAG |
| **HSP60-F(KpnI)** | GGGGTACCCTTCATGCCTCCAACGGCTACTAT |
| **HSP60-R(KpnI)** | GGGGTACCGCGAAAAGCCAATTAGTGTG |
| **HSP10-F(SphI)** | ACATGCATGCGCTACGCAGGCTGCACAATTA |
| **HSP10-R(SphI)** | ACATGCATGCGGAATCTGTGTATATTACTGCATCTAG |

**Table S6 Gradient profile of LC-MS/MS**

| **Steps** | **Time (min)** | **Solution A (vol. %)** | **Solution B (vol. %)** |
| --- | --- | --- | --- |
| **1** | 0 | 100 | 0 |
| **2** | 0.33 | 100 | 0 |
| **3** | 10.33 | 90 | 10 |
| **4** | 20.33 | 40 | 60 |
| **5** | 22.00 | 0 | 100 |
| **6** | 25.00 | 0 | 100 |
| **7** | 25.5 | 100 | 0 |
| **8** | 30 | 100 | 0 |

## Sequence of the codon-optimized *cbbM* (1380 bps):

ATGGACCAATCTGCTAGATACGCTGACTTGTCTTTGAAGGAAGAAGACTTGATCAAGGGTGGTAGACACATCTTGGTTGCTTACAAGATGAAGCCAAAGTCTGGTTACGGTTACTTGGAAGCTGCTGCTCACTTCGCTGCTGAATCTTCTACTGGTACTAACGTTGAAGTTTCTACTACTGACGACTTCACTAAGGGTGTTGACGCTTTGGTTTACTACATCGACGAAGCTTCTGAAGACATGAGAATCGCTTACCCATTGGAATTGTTCGACAGAAACGTTACTGACGGTAGATTCATGTTGGTTTCTTTCTTGACTTTGGCTATCGGTAACAACCAAGGTATGGGTGACATCGAACACGCTAAGATGATCGACTTCTACGTTCCAGAAAGATGTATCCAAATGTTCGACGGTCCAGCTACTGACATCTCTAACTTGTGGAGAATCTTGGGTAGACCAGTTGTTAACGGTGGTTACATCGCTGGTACTATCATCAAGCCAAAGTTGGGTTTGAGACCAGAACCATTCGCTAAGGCTGCTTACCAATTCTGGTTGGGTGGTGACTTCATCAAGAACGACGAACCACAAGGTAACCAAGTTTTCTGTCCATTGAAGAAGGTTTTGCCATTGGTTTACGACGCTATGAAGAGAGCTCAAGACGACACTGGTCAAGCTAAGTTGTTCTCTATGAACATCACTGCTGACGACCACTACGAAATGTGTGCTAGAGCTGACTACGCTTTGGAAGTTTTCGGTCCAGACGCTGACAAGTTGGCTTTCTTGGTTGACGGTTACGTTGGTGGTCCAGGTATGGTTACTACTGCTAGAAGACAATACCCAGGTCAATACTTGCACTACCACAGAGCTGGTCACGGTGCTGTTACTTCTCCATCTGCTAAGAGAGGTTACACTGCTTTCGTTTTGGCTAAGATGTCTAGATTGCAAGGTGCTTCTGGTATCCACGTTGGTACTATGGGTTACGGTAAGATGGAAGGTGAAGGTGACGACAAGATCATCGCTTACATGATCGAAAGAGACGAATGTCAAGGTCCAGTTTACTTCCAAAAGTGGTACGGTATGAAGCCAACTACTCCAATCATCTCTGGTGGTATGAACGCTTTGAGATTGCCAGGTTTCTTCGAAAACTTGGGTCACGGTAACGTTATCAACACTGCTGGTGGTGGTTCTTACGGTCACATCGACTCTCCAGCTGCTGGTGCTATCTCTTTGAGACAATCTTACGAATGTTGGAAGCAAGGTGCTGACCCAATCGAATTCGCTAAGGAACACAAGGAATTCGCTAGAGCTTTCGAATCTTTCCCAAAGGACGCTGACAAGTTGTTCCCAGGTTGGAGAGAAAAGTTGGGTGTTCACTCTTAA

## Sequence of the codon-optimized *cbbL1* (1458 bps):

ATGAACGCTCCAGAATCTGTTCAAGCTAAGCCAAGAAAGAGATACGACGCTGGTGTTATGAAGTACAAGGAAATGGGTTACTGGGACGGTGACTACGAACCAAAGGACACTGACTTGTTGGCTTTGTTCAGAATCACTCCACAAGACGGTGTTGACCCAGTTGAAGCTGCTGCTGCTGTTGCTGGTGAATCTTCTACTGCTACTTGGACTGTTGTTTGGACTGACAGATTGACTGCTTGTGACATGTCTGTTCAAGGTTTGAGAGTTGACCCAGTTCCAAACAACCCAGAACAATTCTTCTGTTACGTTGCTTACGACTTGTCTTTGTTCGAAGAAGGTTCTATCGCTAACTTGACTGCTTCTATCATCGGTAACGTTTTCTCTTTCAAGCCAATCAAGGCTGCTAGATTGGAAGACATGAGATTCCCAGTTGCGTACGTTAAGACATTCGCTGGTCCATCGACTGGTATCATCGTTGAAAGAGAAAGATTGGACAAGTTCGGTAGACCATTGTTGGGTGCTACTACTAAGCCAAAGTTGGGTTTGTCTGGTAGAAACTACGGTAGAGTTGTTTACGAAGGTTTGAAGGGTGGTTTGGACTTCATGAAGGACGACGAAAACATCAACTCTCAACCATTCATGCACTGGAGAGACAGATTCTTGTTCGTTATGGACGCTGTTAACAAGGCTTCTGCTGCTACTGGTGAAGTTAAGGGTTCTTACTTGAACGTTACTGCTGGTACTATGGAAGAAATGTACAGAAGAGCTGAATTCGCTAAGTCTTTGGGTTCTGTTATCATCATGATCGACTTGATCGTTGGTTGGACTTGTATCCAATCTATGTCTAACTGGTGTAGACAAAACGACATGATCTTGCACTTGCACAGAGCTGGTCACGGTACTTACACTAGACAAAAGAACCACGGCGTTAGCTTCAGAGTTATCGCGAAGTGGTTGAGATTGGCTGGTGTTGACCACATGCACACTGGTACTGCTGTTGGTAAGTTGGAAGGTGACCCATTGACTGTTCAAGGTTACTACAACGTTTGTAGAGACGCTTACACTCACGCTGACTTGTCTAGAGGTTTGTTCTTCGATCAAGACTGGGCTAGCTTGAGAAAGGTTATGCCAGTTGCTTCTGGTGGTATCCACGCTGGCCAAATGCACCAGTTGATCAGCTTGTTCGGTGACGACGTTGTTTTGCAATTCGGTGGTGGTACTATCGGTCACCCACAAGGTATCCAAGCTGGTGCTACTGCTAACAGAGTTGCTTTGGAAGCTATGGTTTTGGCTAGAAACGAAGGTAGAGACATCTTGAACGAAGGTCCAGAAATCTTGAGAGACGCTGCTAGATGGTGTGGTCCATTGAGAGCTGCTTTGGACACTTGGGGTGACATCTCTTTCAACTACACTCCAACTGACACTTCTGACTTCGCTCCAACTGCTTCTGTTGCTTAA

## Sequence of the codon-optimized *cbbS1* (420 bps):

ATGAGAATCACTCAAGGTACTTTCTCTTTCTTGCCAGACTTGACTGACGCTCAAATCACTTCTCAATTGGAATACTGTTTGAACCAAGGTTGGGCTGTTGGTATCGAATACACTGACGACCCACACCCAAGAAACACTTACTGGGAAATGTTCGGTTTGCCAATGTTCGACTTGAGAGACGCTGCTGGTATCTTGTTGGAAATCAACAACGCTAGATCTACTTTCCCAAACCACTACATCAGAGTTACTGCTTTCGACTCTACTCACACTGTTGAATCTGTTGTTATGTCTTTCATCGTTAACAGACCAGCTGACGAACCAGGTTTCAGATTGGTTAGACAAGAAGAACCAGGTAGAACTATGAGATACTCTATCGAATCTTACGCTGTTCAAGCTAGACCAGAAGGTTCTAGATACTAA

## Sequence of the codon-optimized *sPRK* (1059 bps):

ATGTCTCAACAACAAACTATCGTTATCGGTTTGGCTGCTGACTCTGGTTGTGGTAAGTCTACTTTCATGAGAAGATTGACTAGTGTCTTCGGGGGTGCTGCTGAACCACCAAAGGGTGGTAACCCAGACTCTAACACTTTGATCTCTGACACTACTACTGTTATCTGTTTGGACGACTTCCACTCTTTGGACAGAAACGGTAGAAAGGTTGAAAAGGTTACTGCTTTGGACCCAAAGGCTAACGACTTCGACTTGATGTACGAACAAGTTAAGGCTTTGAAGGAAGGTAAGGCTGTTGACAAGCCAATCTACAACCACGTTTCTGGTTTGTTGGACCCACCAGAATTGATCCAACCACCAAAGATCTTGGTTATCGAAGGTTTGCACCCAATGTACGACGCTAGAGTTAGAGAATTGTTGGACTTCTCTATCTACTTGGACATCTCTAACGAAGTTAAGTTCGCTTGGAAGATCCAAAGAGACATGAAGGAAAGAGGTCACTCTTTGGAATCTATCAAGGCTTCTATCGAATCTAGAAAGCCAGACTTCGACGCTTACATCGACCCACAAAAGCAACACGCTGACGTTGTTATAGAAGTGCTGCCAACTGAATTGATCCCAGACGACGACGAAGGTAAGGTTTTGAGAGTTAGAATGATCCAAAAGGAAGGTGTTAAGTTCTTCAACCCAGTTTACTTGTTCGACGAAGGTTCTACTATCTCTTGGATCCCATGTGGTAGAAAGTTGACTTGTTCTTACCCAGGTATCAAGTTCTCTTACGGTCCAGACACTTTCTACGGTAACGAAGTTACTGTTGTTGAAATGGACGGTATGTTCGACAGATTGGACGAATTGATCTACGTTGAATCTCACTTGTCTAACTTGTCTACTAAGTTCTACGGTGAAGTTACTCAACAAATGTTGAAGCACCAAAACTTCCCAGGTTCTAACAACGGTACTGGTTTCTTCCAAACTATCATCGGTTTGAAGATCAGAGACTTGTTCGAACAATTGGTTGCTTCTAGATCTACTGCTACTGCTACTGCTGCTAAGGCTTAA

## Sequence of the codon-optimized *cfxP1* (879 bps):

ATGTCTGAAAGATACCCAATCATCGCTATCACTGGTTCTTCTGGTGCTGGTACTACTTCTGTTACTAGAACTTTCGAAAACATCTTCAGAAGAGAAGGTGTTAAGTCTGTTGTTATAGAGGGTGACAGCTTCCACAGATACGACAGAGCTGAAATGAAGGTTAAGATGGCTGAAGCTGAAAGAACTGGTAACATGAACTTCTCTCACTTCGGTGAAGAAAACAACTTGTTCGGTGAATTGGAAAACTTGTTCAGATCTTACGCTGAAACTGGTACTGGTATGCACAGACACTACTTGCACTCTCCAGAAGAAGCTGCTCCATTCGGTCAAGAACCAGGTACTTTCACTCAATGGGAACCATTGCCAGCTGACACTGACTTGTTGTTCTACGAAGGTTTGCACGGTGGTGTTGTTACTGACTCTGTTAACGTTGCTCAATACCCAAACTTGTTGATCGGTGTTGTTCCAGTTATCAACTTGGAATGGATCCAAAAGTTGTGGAGAGACAAGAAGCAAAGAGGTTACTCTACTGAAGCTGTTACTGACACTATCTTGAGAAGAATGCCAGACTACGTTAACTACATCTGTCCACAATTCTCTAGAACTCACGTTAACTTCCAAAGAGTTCCATGTGTTGACACTTCTAACCCATTCATCTCTAGAGAAATCCCAGCTCCAGACGAATCTATGGTTGTTATCAGATTCGCTAACCCAAAGGGTATCGACTTCCAATACTTGTTGTCTATGATCCACGACTCTTTCATGTCTAGAGCTAACACTATCGTTGTTCCAGGTGGTAAGATGGAATTGGCTATGCAATTGATCTTCACTCCATTCGTTTTGAGAATGATGGAAAGAAGAAAGAGAGCTGCTCAATAA

## Sequence of the codon-optimized *GroEL* (1647 bps):

ATGGCTGCTAAGGACGTTAAGTTCGGTAACGACGCTAGAGTTAAGATGTTGAGAGGTGTTAACGTTTTGGCTGACGCTGTTAAGGTTACTTTGGGTCCAAAGGGTAGAAACGTTGTTTTGGACAAGTCTTTCGGTGCTCCAACTATCACTAAGGACGGTGTTTCTGTTGCTAGAGAAATCGAATTGGAAGACAAGTTCGAAAACATGGGTGCTCAAATGGTTAAGGAAGTTGCTTCTAAGGCTAACGACGCTGCTGGTGACGGTACTACTACTGCTACTGTTTTGGCTCAAGCTATCATCACTGAAGGTTTGAAGGCTGTTGCTGCTGGTATGAACCCAATGGACTTGAAGAGAGGTATCGACAAGGCTGTTACTGCTGCTGTTGAAGAATTGAAGGCTTTGTCTGTTCCATGTTCTGACTCTAAGGCTATCGCTCAAGTTGGTACTATCTCTGCTAACTCTGACGAAACTGTTGGTAAGTTGATCGCTGAAGCTATGGACAAGGTTGGTAAGGAAGGTGTTATCACTGTTGAAGACGGTACTGGTTTGCAAGACGAATTGGACGTTGTTGAAGGTATGCAATTCGACAGAGGTTACTTGTCTCCATACTTCATCAACAAGCCAGAAACTGGTGCTGTTGAATTGGAATCTCCATTCATCTTGTTGGCTGACAAGAAGATCTCTAACATCAGAGAAATGTTGCCAGTTTTGGAAGCTGTTGCTAAGGCTGGTAAGCCATTGTTGATCATCGCTGAAGACGTTGAAGGTGAAGCTTTGGCTACTGCTGTTGTTAACACTATCAGAGGTATCGTTAAGGTTGCTGCTGTTAAGGCTCCAGGTTTCGGTGACAGAAGAAAGGCTATGTTGCAAGATATCGCTACTCTCACTGGTGGTACTGTTATCTCTGAAGAAATCGGTATGGAATTGGAAAAGGCTACTTTGGAAGACTTGGGTCAAGCTAAGAGAGTTGTTATCAACAAGGACACTACTACTATCATCGACGGTGTTGGTGAAGAAGCTGCTATCCAAGGTAGAGTTGCTCAAATCAGACAACAAATCGAAGAAGCTACTTCTGACTACGACAGAGAAAAGTTGCAAGAAAGAGTTGCTAAGTTGGCTGGTGGTGTTGCTGTTATCAAGGTTGGTGCTGCTACTGAAGTTGAAATGAAGGAAAAGAAGGCTAGAGTTGAAGACGCTTTGCACGCTACTAGAGCTGCTGTTGAAGAAGGTGTTGTTGCTGGTGGTGGTGTTGCTTTGATCAGAGTTGCTTCTAAGTTGGCTGACTTGAGAGGTCAAAACGAAGACCAAAACGTTGGTATCAAGGTTGCTTTGAGAGCTATGGAAGCTCCATTGAGACAAATCGTTTTGAACTGTGGTGAAGAACCATCTGTTGTTGCTAACACTGTTAAGGGTGGTGACGGTAACTACGGTTACAACGCTGCTACTGAAGAATACGGTAACATGATCGACATGGGTATCTTGGACCCAACTAAGGTTACTAGATCTGCTTTGCAATACGCTGCTTCTGTTGCTGGTTTGATGATCACTACTGAATGTATGGTTACTGACTTGCCAAAGAACGACGCTGCTGACTTGGGTGCTGCTGGTGGTATGGGTGGTATGGGTGGTATGGGTGGTATGATGTAA

## Sequence of the codon-optimized *GroES*(294 bps):

ATGAACATCAGACCATTGCACGACAGAGTTATCGTTAAGAGAAAGGAAGTTGAAACTAAGTCTGCTGGTGGTATCGTTTTGACTGGTTCTGCTGCTGCTAAGTCTACTAGAGGTGAAGTTTTGGCTGTTGGTAACGGTAGAATCTTGGAAAACGGTGAAGTTAAGCCATTGGACGTTAAGGTTGGTGACATCGTTATCTTCAACGACGGTTACGGTGTTAAGTCTGAAAAGATCGACAACGAAGAAGTTTTGATCATGTCTGAATCTGACATCTTGGCTATCGTTGAAGCTTAA

## Sequence of the codon-optimized *HSP60* (1647bps):

ATGGAATTGAAATTCGGTGTAGAAGGAAGAGCCTCCCTTCTTAAGGGTGTCGAAACTTTAGCTGAAGCGGTTGCTGCTACTTTGGGTCCAAAGGGTAGAAACGTTTTAATCGAACAGCCTTTCGGTCCTCCAAAGATTACTAAGGATGGTGTTACAGTTGCCAAATCTATTGTGTTGAAGGACAAGTTTGAAAATATGGGTGCCAAGTTACTACAAGAAGTTGCCTCCAAAACCAATGAGGCTGCTGGTGACGGTACTACTTCTGCTACTGTCTTAGGTAGAGCCATCTTCACAGAATCCGTCAAAAATGTCGCCGCTGGTTGTAACCCTATGGATTTGAGAAGGGGTTCTCAAGTTGCAGTTGAAAAAGTGATTGAATTTTTGAGCGCCAACAAGAAAGAAATTACCACATCTGAGGAAATTGCTCAAGTAGCAACCATTTCTGCCAATGGGGACTCTCATGTTGGTAAGTTACTAGCTTCAGCTATGGAAAAGGTTGGAAAAGAAGGTGTCATCACTATCAGAGAAGGTAGAACATTGGAAGATGAACTTGAGGTTACTGAAGGTATGAGGTTTGATCGTGGTTTTATTTCTCCATACTTCATCACTGATCCAAAGTCGAGCAAGGTGGAATTTGAAAAGCCATTGCTATTGTTGAGTGAAAAGAAAATTTCTTCCATTCAAGATATCTTGCCAGCTTTGGAAATTTCCAATCAAAGCAGAAGACCTTTGTTGATCATTGCTGAAGATGTTGACGGTGAAGCTCTTGCGGCTTGTATTTTGAACAAGTTGAGGGGTCAAGTTAAGGTTTGTGCTGTGAAGGCGCCTGGTTTCGGTGATAATAGAAAGAATACAATTGGTGATATTGCAGTCTTGACGGGCGGTACTGTTTTTACTGAGGAGTTGGATTTGAAACCAGAACAATGTACCATAGAAAACTTGGGTTCTTGTGACTCTATTACCGTTACTAAGGAAGACACCGTTATCCTGAACGGTAGTGGTCCAAAGGAAGCTATTCAAGAGAGAATTGAACAAATCAAAGGCTCCATCGACATTACCACCACAAATTCATATGAGAAGGAGAAACTGCAAGAGCGTTTGGCCAAATTGTCCGGGGGTGTTGCTGTCATCAGGGTCGGTGGTGCATCTGAAGTTGAAGTTGGTGAAAAGAAGGACCGTTACGATGATGCTTTGAACGCTACCAGAGCTGCAGTTGAGGAAGGTATCTTGCCAGGTGGTGGTACTGCCTTAGTGAAGGCATCTAGAGTTTTGGATGAAGTTGTTGTCGACAATTTCGATCAAAAATTGGGTGTCGATATCATAAGAAAGGCCATTACAAGACCAGCCAAGCAGATCATTGAAAACGCTGGTGAAGAAGGTTCAGTTATCATCGGCAAATTGATTGATGAATATGGTGATGATTTTGCCAAGGGTTACGATGCCTCTAAGTCAGAATACACCGACATGTTAGCCACTGGTATCATCGATCCATTTAAAGTGGTTAGATCCGGTTTAGTTGATGCTTCTGGTGTTGCCTCACTATTAGCTACTACCGAAGTTGCTATTGTTGATGCCCCAGAACCACCAGCAGCTGCTGGCGCTGGTGGTATGCCAGGTGGTATGCCAGGAATGCCAGGTATGATGTAA

## Sequence of the codon-optimized *HSP10* (297 bps):

ATGTCTATCGTTCCATTGATGGACCGTGTCCTTGTCCAAAGAATCAAGGCACAAGCAAAGACAGCATCCGGGTTGTATTTACCTGAAAAGAACGTGGAGAAGTTAAACCAAGCTGAAGTTGTTGCCGTAGGCCCGGGCTTTACTGATGCTAATGGTAATAAGGTTGTTCCTCAAGTTAAAGTTGGTGACCAAGTTTTGATTCCACAGTTTGGTGGTTCTACCATTAAATTGGGTAACGACGATGAAGTTATTCTTTTCAGGGACGCTGAAATCCTGGCTAAGATTGCCAAGGACTAA

# Reference

1. Fan, L. H., Zhang, Z. J., Yu, X. Y., Xue, Y. X., & Tan, T. W. Self-surface assembly of cellulosomes with two miniscaffoldins on saccharomyces cerevisiae for cellulosic ethanol production. *Proc. Natl. Acad. Sci. USA***109**, 13260-5 (2012).
2. Fan, L. H. *et al*. Engineering yeast with bifunctional minicellulosome and cellodextrin pathway for co-utilization of cellulose-mixed sugars. *Biotechnol. Biofuels* **9**, 1-11(2016).
